# A Review of Non-Laser and Laser Machining for Through-Glass via Fabrication

**DOI:** 10.3390/mi17070796

**Published:** 2026-06-29

**Authors:** Yong Zhang, Keke Zhang, Yapeng Xu, Wenjun Tong, Junfeng Wang, Wuyi Ming

**Affiliations:** 1 School of Intelligent Manufacturing Equipment, Guangdong Mechanical & Electrical Polytechnic, Guangzhou 510550, China; zhangyong904@163.com; 2 Mechanical and Electrical Engineering Institute, Zhengzhou University of Light Industry, Zhengzhou 450002, China; 15515989873@163.com; 3 Guangdong HUST Industrial Technology Research Institute, Huazhong University of Science and Technology, Dongguan 523808, China; 4 School of Aerospace Engineering, Huazhong University of Science and Technology, Wuhan 430074, China; tongwj@hust.edu.cn; 5 NanYang DTECH Co., Ltd., Nanyang 473500, China; wangjunfeng@ucanrobot.com

**Keywords:** through-glass vias (TGVs), advanced packaging, non-laser processing, laser processing, hole quality, uniformity and reliability

## Abstract

As semiconductor packaging technology evolves from two-dimensional to three-dimensional integration, the through-glass via (TGV) technique, as a core interconnect method in advanced packaging, is emerging as a strong candidate to replace through-silicon vias (TSVs) and plated through-holes (PTHs) in organic substrates. Glass substrates offer excellent electrical insulation, low dielectric loss, tunable thermal expansion coefficients, and the potential for large-scale panel-level manufacturing. However, issues related to TGV hole quality, metallization uniformity, and thermomechanical reliability remain key bottlenecks limiting their large-scale industrialization. This investigation provides a comparative review of non-laser and laser machining for TGVs to address the above problems. First, the technical background and core advantages of TGVs are outlined. Second, this study details non-laser processing methods, including sandblasting erosion, mechanical drilling, the photosensitive glass method, electrochemical discharge machining (ECDM), deep reactive ion etching (DRIE), and others. Third, laser processing methods, covering laser ablation drilling, laser-induced deep etching (LIDE), femtosecond laser-assisted wet etching and others, are given focus. Moreover, this study analyzes typical applications of TGVs in 3D/2.5D packaging, MEMS devices, optoelectronic integration, and others. In addition, the machining processes of non-laser and laser-based TGVs, such as mechanical machining, ECDM, and LIDE, are compared, and key process challenges, technical trade-offs, and reliability failure mechanisms are discussed. Finally, this review looks ahead to future trends, aiming to provide a systematic technical reference for researchers in the TGV field.

## 1. Introduction

As semiconductor processes approach their physical limits, the industry’s focus is shifting from transistor miniaturization to system-level packaging integration. 3D stacking, heterogeneous integration, and 2.5D/3D packaging enable vertical interconnections between dies manufactured using different processes, shortening signal paths and reducing power consumption [1,2,3]. This requires substrates to support high-density vertical interconnections while maintaining low loss and high reliability during the transmission of high-speed signals and power.

For a long time, the through-silicon via (TSV) technique has been widely utilized in 2.5D/3D packaging, but its inherent limitations are becoming increasingly apparent. Silicon has a high dielectric constant (εr ≈ 11.7) and loss tangent (tanδ ≈ 0.01), resulting in signal transmission losses that increase significantly with rising frequency, a problem that is particularly severe in the millimeter-wave band [1,4,5]. Furthermore, the significant mismatch in the coefficient of thermal expansion (CTE) between silicon and typical packaging materials can easily lead to interface stress accumulation and potential failure. At the same time, traditional organic substrates are limited by dimensional stability and wiring density [6], making it difficult to meet the ultra-high-density interconnect requirements of advanced applications such as AI and high-performance computing.

**Figure 1:**
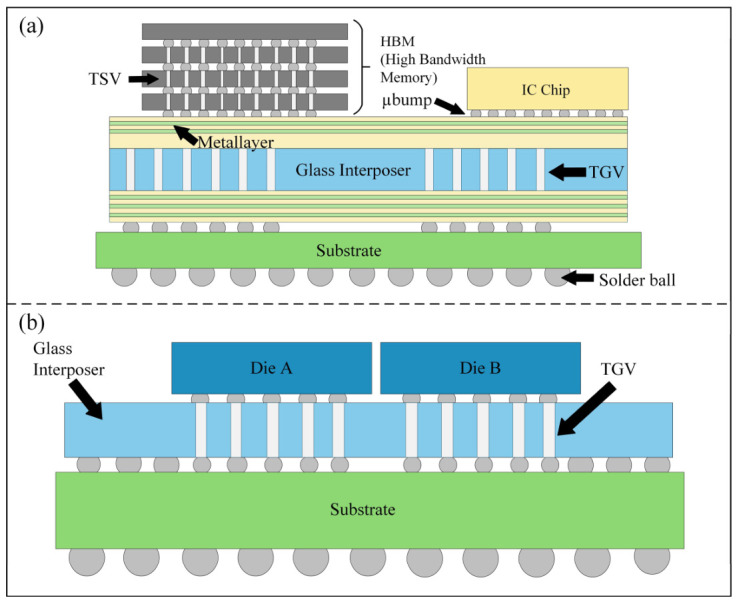
TGV packaging solutions in advanced semiconductor packages. (**a**) TGV glass interposers in a 2.5D advanced semiconductor package; (**b**) TGV glass core boards in a multi-die module package.

Glass substrates and the through-glass via (TGV) technique have consequently emerged as key solutions [7,8]. By creating copper-filled microvias that penetrate the glass, these techniques enable electrical connections between chips to travel the shortest possible vertical distance. Additionally, the dielectric properties and high-frequency characteristics of glass itself outperform those of traditional organic substrates, making it capable of meeting the stringent bandwidth and energy efficiency requirements of high-performance computing (HPC) and artificial intelligence (AI) chips. Current TGV solutions have evolved into various interconnect architectures and can be integrated into more complex packaging designs. Consequently, packaging solutions based on TGVs are primarily developing along two paths: TGV glass interposers and TGV glass core boards, as shown in Figure 1. For example, the excellent dimensional stability of glass enables the scaling of fine features, and the through-hole density it supports can be more than ten times that of organic substrates. Glass suppliers can provide large-size panels (even exceeding 510 × 515 mm^2^), and this panel-level manufacturing capability is expected to result in lower manufacturing costs than wafer-level solutions [9,10].

Beyond their role as interconnect substrates, TGV-based glass platforms have been increasingly explored in 2.5D/3D packaging, MEMS device packaging, optoelectronic integration, co-packaged optics, Micro-LED displays, biomedical electronics, quantum devices, and millimeter-wave automotive electronics. In these application scenarios, the advantages of glass, including low dielectric loss, adjustable CTE, optical transparency, chemical stability, and high dimensional stability, are directly linked to key fabrication requirements such as high via density, smooth sidewalls, high aspect ratio, hermetic sealing, and reliable metallization. Therefore, an application-oriented understanding of TGVs is necessary for evaluating different machining methods and selecting suitable processes for advanced packaging systems.

In terms of market demand, the TGV substrate market was valued at approximately $60 million in 2022 and is projected to grow to $480.5 million by 2029, with a compound annual growth rate (CAGR) of 34.2% over the forecast period, clearly demonstrating the rapid pace of its industrialization [11]. However, the implementation of the TGV technique is not without challenges. The inherently brittle nature of glass makes it highly susceptible to cracking due to stress concentration, which not only undermines the structural integrity of the through-holes but also directly threatens the long-term reliability of the package [12,13]. During the hole-forming process, controlling hole dimensions, managing sidewall roughness, suppressing microcracks, and ensuring compatibility with subsequent metallization processes collectively constitute the core technical challenges of TGV manufacturing. These challenges, in turn, affect the uniformity of subsequent copper filling, the control of voids and defects, and the reliability of adhesion to the glass surface.

The range of TGV processing methods is extremely diverse, spanning from traditional mechanical removal to advanced laser modification. Based on the energy source and the core mechanism of material removal, these techniques can be clearly divided into two major categories: non-laser machining and laser machining. This distinction is made because the employment of lasers directly determines the limits of processing precision, the extent of the heat-affected zone, and the complexity of process integration. Non-laser machining primarily includes sandblasting erosion [14], mechanical drilling [15], photoresist-coated glass [16], electrochemical discharge machining (ECDM) [17] and others [18,19]; these methods rely mainly on mechanical impact, discharge ablation, or chemical etching to form the desired features. Laser machining, on the other hand, encompasses methods such as laser ablation drilling, laser-induced deep etching (LIDE), and ultrafast laser-assisted wet etching (by femtosecond laser). For clarity, representative non-laser and laser-based TGV fabrication methods are further summarized in Table 1, with emphasis on their processing principles, main features, limitations, and typical applications.

In recent years, several reviews have summarized TGV advancements. For example, Ma et al. [20] focused on process development and reliability, with particular emphasis on thermal stress-induced interconnect failures. Other reviews have specifically analyzed the thermomechanical reliability of glass substrates and TGVs, identifying the brittleness and crack resistance of glass as key obstacles to industrialization [9]. Beom et al. [11], on the other hand, centered on summarizing process advancements in drilling, functional layer deposition, and copper filling. Most of these studies are organized by specific process routes and have not yet addressed the issue from the perspective of material removal mechanisms. They have not categorized non-laser methods (such as sandblasting, electrical discharge machining, and dry etching) and laser methods (such as laser ablation, LIDE, and ultrafast laser-assisted etching) into two broad categories, nor have they conducted systematic comparisons regarding hole quality, metallization uniformity, and thermomechanical reliability. This review distinguishes itself by adopting a “non-laser processing vs. laser processing” comparative framework, integrating the three core metrics mentioned above throughout the text. It examines the differences between the two categories of processes—under the same metrics—in terms of hole precision, sidewall condition, seed layer coverage, fill defect rate, and interfacial behavior under thermal stress mismatch, aiming to provide a comparative perspective that aligns more closely with industrial selection logic.

Thereafter, this study aims to fill this gap by providing a systematic review of the latest advancements in TGV processing techniques. Unlike existing reviews, which typically organize content by specific process routes, this investigation clearly categorizes through-hole fabrication methods into two main categories: non-laser machining and laser machining. It then conducts a systematic comparison based on three key criteria: hole quality, metallization uniformity, and thermomechanical reliability. Specifically, Section 1 outlines the background and core advantages of the TGV technique. Section 2 details non-laser machining methods, covering sandblasting erosion, mechanical drilling, the photosensitive glass method, ECDM, deep reactive ion etching (DRIE), and others. Correspondingly, Section 3 focuses on laser machining methods, including laser direct ablation drilling, LIDE, femtosecond laser-assisted wet etching, and others. In addition, Section 4 briefly summarizes representative applications of TGVs and links these application scenarios to corresponding processing requirements. Moreover, Section 5 discusses the two major categories of methods mentioned above, analyzing key process challenges, reliability failure mechanisms, and technical trade-offs one by one. Accordingly, Section 6 looks ahead to future trends, aiming to provide a systematic technical reference for researchers in the TGV field.

## 2. Non-Laser Machining for TGVs

Before the widespread adoption of laser techniques in glass microfabrication, TGV hole formation primarily relied on a range of traditional methods based on mechanical, chemical, and electrical discharge principles. Although these methods have their own limitations in terms of precision, aspect ratio, and production capacity, they laid the technical foundation for the manufacture of through-holes in glass and remain irreplaceable in certain specific applications.

### 2.1. Sandblasting Erosion

**Figure 2:**
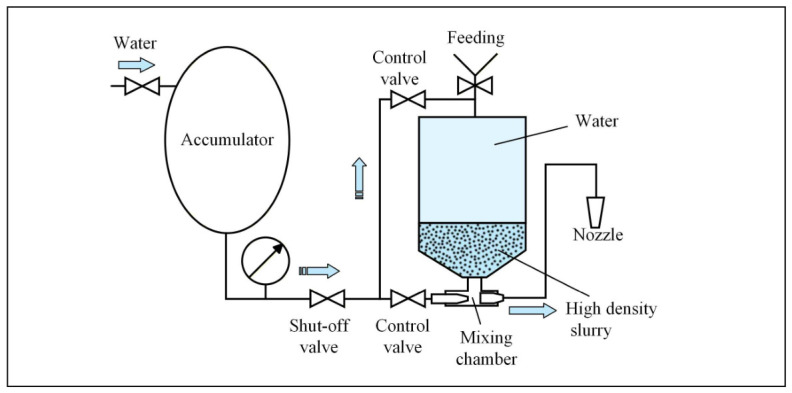
Performance of sandblasting erosion for TGVs: schematic of experimental setup for low-pressure slurry jet micromachining.

Sandblasting is one of the earliest traditional methods utilized for the mass production of glass through-holes. The principle involves using a high-pressure air stream carrying fine abrasive particles to impact the glass surface at high speed, removing material layer by layer through the cutting and impact actions of the abrasive particles, as depicted in Figure 2. Combined with a patterned mask, this enables selective etching to form through-holes. Malou et al. [21] examined the effect of sand particle impact on the thermal shock resistance of soda-lime glass. Experiments showed that the samples with the most severe sandblasting damage exhibited the lowest thermal shock resistance, with a significant decrease in the critical temperature difference. These microcracks may serve as crack initiation sites during thermal cycling in packaging (typically from −40 °C to +125 °C), leading to delamination failure between the copper filler and the glass hole walls, which directly threatens thermomechanical reliability. Ghobeity et al. [22] established a model for predicting the micro-hole machining of glass and polymethyl methacrylate (PMMA) via abrasive jet machining. The hole profiles predicted by this model showed good agreement with both experimental results and computer simulations. When the aspect ratio of PMMA did not exceed 0.6, both shape and depth exhibited good consistency, with a maximum error of 13%. Zheng et al. [23] investigated the effects of mask aperture size, compressed air pressure, and abrasive particle size on the etching morphology of glass micromachining via sandblasting. The results indicated that reducing the abrasive particle size from 30 to 20 μm improved the formed morphology, meeting the etching requirements for a glass through-hole array with a depth of 500 μm. However, the etched aperture size of this method exceeds 700 μm, and the aspect ratio of the glass micropores is less than 1. Qi et al. [24] proposed ultrasonic vibration-assisted abrasive slurry jet (UV-ASJ) processing for K9 glass micropores to overcome the typical “W-shaped” bottom defects in conventional low-pressure ASJ with fine abrasive particles. Their study found that ultrasonic vibration primarily enhanced machining performance by altering particle trajectories in the stagnation zone and modifying the dynamic impact process. Quantitative analysis indicated that the key machining indicators under UV-ASJ conditions were significantly higher than those of conventional ASJ. This work provides new insights for improving hole bottom quality in sandblasting-type methods, demonstrating that ultrasonic assistance can effectively eliminate W-shaped bottoms and achieve flatter hole bottoms. In summary, although sandblasting has played a role in early TGV exploration due to its low equipment costs and ability to process large-area arrays in parallel, its inherent limitations in terms of hole quality, metallization uniformity, and thermomechanical reliability make it difficult to meet the stringent requirements of high-frequency, high-density interconnects.

### 2.2. Mechanical Drilling

**Figure 3:**
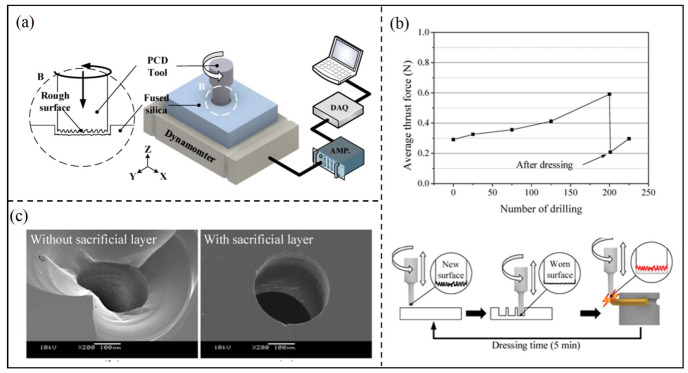
Principle and performance of fused-silica microdrilling using EDMed PCD tools [27]; (**a**) schematic of the microdrilling setup and abrasive material-removal action of the rough PCD tool surface; (**b**) variation in average thrust force with the number of drilling operations and the EDM dressing process used to regenerate the worn tool surface; (**c**) SEM images of the exit sides of through-holes drilled without and with a sacrificial layer.

Mechanical drilling is the most established method for through-hole fabrication in traditional PCB manufacturing and has also been attempted for the preparation of through-holes in glass substrates. This method employs microdrills (typically made of cemented carbide or diamond-coated) to cut through the glass material via high-speed rotation of the spindle. In mechanical drilling processes for organic substrates, through-holes with diameters ranging from 0.2 mm to 1 mm can be achieved, with extremely high production capacity. However, the high hardness and brittleness of glass pose significant challenges for mechanical drilling. Hof et al. [25] reviewed micro-hole drilling technologies for glass and found that the hole diameter ranges for conventional mechanical drilling and ultrasonic drilling were approximately 100 μm–1 mm and 10 μm–5 mm, respectively. Ultrasonic drilling achieved a surface roughness (Ra) of approximately 0.2–2 μm, outperforming conventional mechanical drilling, and its aspect ratio could reach up to 10. They found that the primary drawbacks of mechanical drilling were edge chipping and taper, while ultrasonic drilling was capable of producing straight-walled and high-aspect-ratio through-holes. Notably, hole wall roughness directly affects the quality of subsequent metallization, as rough walls hinder the uniform deposition of the seed layer. Furthermore, microcracks and subsurface damage act as crack initiation sites during thermal cycling, directly threatening interconnect reliability. Ma et al. [26] investigated the formation mechanism of hole damage during optical glass drilling and preliminarily explored the damage-suppression effects of rotary ultrasonic drilling (RUD) and rotary ultrasonic peck drilling (RUPD) processes. Experimental results indicated that “local chipping” was the primary form of damage at the hole entrance, while “global spalling” and “discontinuous fragmentation” combined to characterize damage at the hole exit. RUD effectively suppressed hole damage, with maximum entrance chipping dimensions and maximum exit spalling dimensions amounting to 15–50% and 45–65% of those observed in conventional drilling, respectively. RUPD promoted the ejection of swarf and detached abrasive particles, resulting in reduced exit damage. Lee et al. [27] investigated the mechanical microdrilling of fused silica using polycrystalline diamond (PCD) tools fabricated by electrical discharge machining. A Ø300 μm PCD tool with an electrically generated rough surface was rotated at 50,000 rpm to drill 0.5 mm thick fused silica, while the drilling forces were continuously measured using a dynamometer, as shown in Figure 3a. The rough craters and exposed diamond grains on the tool surface acted as abrasive cutting edges during material removal. As the number of drilled holes increased, tool wear gradually smoothed the cutting surface, causing the average thrust force to increase from approximately 0.3 N to 0.6 N after 200 drilling operations. A 5 min EDM dressing process regenerated the rough tool surface and reduced the thrust force to approximately 0.2 N, thereby restoring the machining performance of the tool, as shown in Figure 3b. For through-hole drilling, a sacrificial fused-silica layer was introduced beneath the workpiece to support the hole exit and suppress tensile-stress-induced fracture. Compared with drilling without the sacrificial layer, this method substantially reduced exit-side cracking and edge chipping, as shown in Figure 3c. These measures improve through-hole integrity, tool serviceability, and machining consistency, providing a favorable basis for subsequent TGV metallization and reliable interconnection. Chen et al. [28] proposed an innovative grinding–drilling hybrid machining technique. This method integrated a load cell to monitor drilling force in real time, effectively suppressing microcrack formation during machining. It increased tool life by 30 times and achieved high-precision, low-damage microvia machining, ensuring excellent hole quality. Additionally, Kim et al. [29] developed a machine vision-based monitoring system for online monitoring of the microdrilling process to meet the demand for high-quality microvias in optical devices. In the TGV applications, advanced packaging requires through-hole diameters smaller than 100 μm and finer pitch, which exceeds the capabilities of most methods of traditional mechanical drilling. However, it is worth noting that mechanical drilling still holds value in the fabrication of larger-sized through-holes on glass substrates (such as those employed for fluid channels or structural positioning holes).

### 2.3. Photosensitive Glass Method

**Figure 4:**
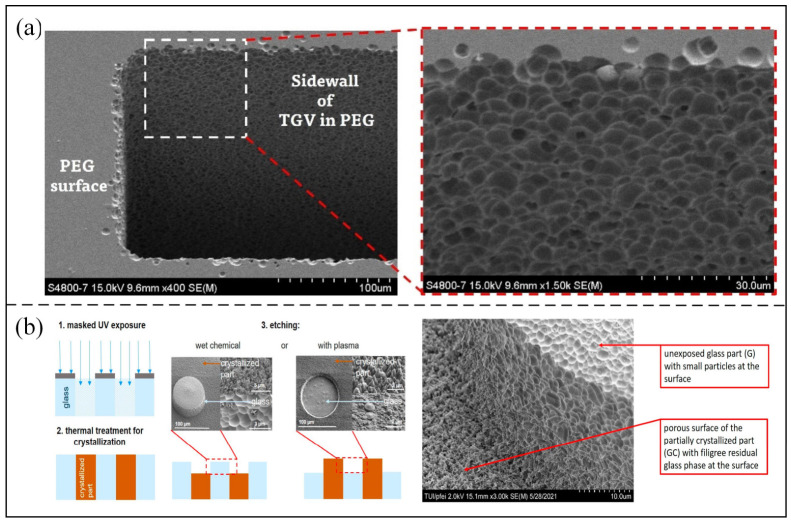
Principle and performance of photosensitive glass method for TGVs; (**a**) Left, SEM morphology of the TGV sidewall in photoetchable glass (PEG); right, enlarged view of the porous sidewall surface [31]; (**b**) left, principle of photosensitive glass under wet chemical etching and plasma etching; right, SEM image of the transition area under wet chemical etching [32].

The photosensitive glass method is a unique pore-forming technique that utilizes the inherent photosensitive properties of glass materials, and its principle shares similarities with traditional photolithography processes. When exposed to light of a specific wavelength, photosensitive glass reacts and undergoes permanent structural changes following heat treatment [30]. The advantage of this method lies in its high precision and consistency, comparable to photolithography processes. Since the exposure pattern is defined by a photomask, the positional accuracy of the through-holes can reach the submicron level typical of photolithography. Chung et al. [31] fabricated continuous TGV structures in photoetchable glass through UV exposure, heat development, and anisotropic HF wet etching, as shown in Figure 4a. A 420 μm-thick glass substrate was etched in a 10% HF solution for 450 s at an etching rate of approximately 60 μm/min, followed by Ti/Cu sputtering to metallize the TGV sidewalls and connect the top and bottom conductive layers. The fabricated substrate-integrated waveguide exhibited an average insertion loss of 2.53 ± 0.55 dB and a return loss better than 13.86 dB over 26.5–40 GHz. This study demonstrates the suitability of photoetchable glass TGVs for wafer-level fabrication of millimeter-wave devices. Brokmann et al. [32] were the first to systematically compare the characteristics of photosensitive glass under wet chemical etching (hydrofluoric acid-based) and plasma etching (CF_4_/H_2_-based), as shown in Figure 4b. They found that during wet chemical etching, the solubility of the crystalline portion was 27 times higher than that of the unexposed glass; during plasma etching, the dissolution rate of the glassy component was approximately 2.5 times that of the partially crystalline component. This reversal implies that plasma etching tends to “preserve” the crystalline structure while removing the surrounding glass matrix. Consequently, wet chemical etching of photosensitive glass can fabricate through-hole structures with high aspect ratios and vertical sidewalls, whereas plasma etching may be suitable for fabricating “bump”-like microstructures rather than conventional vertical through-holes. Furthermore, photosensitive glass can be modified through UV laser irradiation or maskless modification by researchers. Both processes enable vias with aspect ratios greater than 8 (25 to 35) [8,33,34,35].

These studies indicate that the photosensitive glass method demonstrates potential for high-density interconnects and microsystem integration. However, the primary limitation of this method lies in restricted material selection. This means that only specific photoresist glass formulations are available, and they cannot be applied to the alkali-free glass commonly utilized in industry. Furthermore, the high-temperature crystallization heat treatment step increases process complexity and may cause substrate warping. These factors limit the widespread adoption of the photoresist glass method in high-throughput panel-level manufacturing. But it still holds unique value for prototype devices and small-batch production requiring specific performance characteristics.

### 2.4. Electrochemical Discharge Machining

**Figure 5:**
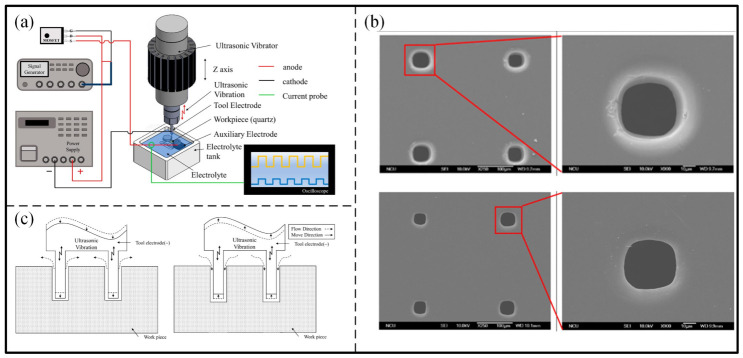
Ultrasonic vibration-assisted ECDM of quartz wafer micro-through-hole arrays [41]: (**a**) experimental setup; (**b**) inlet and outlet morphologies at 44 V; (**c**) electrolyte pumping effect induced by ultrasonic vibration.

The process of ECDM is a specialized microfabrication method that utilizes the thermal energy from electrical discharges to break down and spatter molten glass from the substrate, thereby forming glass through-holes [36,37]. Conventional electrical discharge machining (EDM) requires the workpiece to be conductive, making it impossible to directly machine insulating materials such as glass [38,39]. In the process of ECDM, the glass substrate is immersed in an electrolyte solution (typically an aqueous solution of NaOH or KOH), and a direct-current voltage is applied between the tool electrode (cathode) and the auxiliary electrode (anode). When the voltage exceeds a critical threshold, a bubble layer (hydrogen gas) forms on the surface of the tool electrode due to electrolysis [40]. The insulating properties of this bubble layer induce spark discharges between the electrode and the electrolyte, generating localized temperatures exceeding 3000 °C, which melt and vaporize the glass material to form micropores. Yang et al. [41] employed ultrasonic vibration-assisted electrochemical discharge machining (ECDM) to fabricate micro-through-hole arrays in 70 μm thick quartz wafers. A self-fabricated 2 × 2 tungsten carbide microelectrode array was mounted on an axial ultrasonic vibrator to achieve the simultaneous machining of multiple through-holes, while the machining current was monitored using a current probe and signal acquisition system, as shown in Figure 5a. The reciprocating motion of the tool electrode generated a pumping effect within the narrow machining gaps, alternately expelling and drawing electrolyte into the holes. This promoted electrolyte renewal, facilitated bubble and debris removal, and improved the stability of the insulating gas film and discharge process, as shown in Figure 5c. At a working voltage of 44 V, both the inlet and outlet contours of the micro-through-hole array were relatively smooth, with no obvious unremoved material or outlet fragmentation, as shown in Figure 5b. Under the optimized conditions, the average inlet and outlet diameters were 85.8 and 73.8 μm, respectively, satisfying the target through-hole diameter of 80 ± 8 μm. The improved hole regularity, dimensional consistency, and outlet integrity are beneficial for controlling TGV pitch, suppressing crack initiation, and enhancing the reliability of subsequent metallization and electrical interconnection. Arab et al. [42] investigated the effect of tool electrode surface roughness on the geometric characteristics of through-holes in glass substrates (400 μm thick) during ECDM machining. They found that when using a smooth electrode, the radial overcut of the hole decreased from 160 to 73 μm, the heat-affected zone (HAZ) width narrowed from 87 μm to 48 μm, and the roundness deviations at the entrance and exit of holes decreased. The reduction in radial overcut means that the actual TGV hole diameter is closer to the design value, which is beneficial for pitch control in high-density wiring. The reduction in HAZ width lowers the microcrack density in the material surrounding the holes, laying the foundation for subsequent thermal cycling reliability. Kannojia et al. [43] fabricated a TGV array on 520 μm thick fused silica glass using the ECDM method (~120 μm diameter electrodes) and filled it with electroplated copper. Experimental results showed that the average resistance of the TGV after electroplating was 256 mΩ, with a variation range of < ±8% across different locations on a single wafer. Furthermore, the SEM cross-section after copper electroplating revealed a dense copper fill with no obvious voids. Generally, a resistance variation of less than 10% indicates that the ECDM process, combined with appropriate electroplating parameters, can achieve uniform TGV filling, meeting the basic requirements for signal transmission consistency in integrated packaging. Bajpai et al. [37] proposed a photolithography-free ECDM process to fabricate TGV-based 3D microchannels and MEMS structures, eliminating the need for traditional masking steps. They fabricated an array of through-holes with a diameter of ~450 μm on a 400 μm thick glass substrate, achieving a good entrance roundness deviation and sidewall steepness. Validation results confirmed that the aperture size variation was controlled within a reasonable range during batch testing, and good conductivity was obtained in both square and spiral microstructures, with an average resistance of 2.41 Ω. The photolithography-free process reduces manufacturing costs and process complexity, demonstrating the feasibility of ECDM for MEMS applications ranging from single-hole formation to functional devices. Additionally, other researchers have employed ECDM to fabricate 580 μm through-holes in fused silica substrates to produce 3D spiral inductors [44]. The main advantages of ECDM lie in its simple equipment, low processing costs, and the theoretical ability to process glass of any thickness. However, its control precision of holes is generally poor, and microcracks and thermal damage are present on its sidewalls, with relatively slow processing speeds. These shortcomings mean that ECDM applications in the TGV field are primarily concentrated in the prototyping stage of MEMS devices.

### 2.5. Deep Reactive Ion Etching

Plasma dry etching, particularly DRIE, has been successfully applied in the fabrication of silicon through-holes. The basic principle involves the generation of a high-density plasma from fluorinated gases (such as SF_6_, CF_4_, or CHF_3_) within a vacuum chamber under the influence of a radiofrequency electric field. Fluorine radicals react chemically with SiO_2_ in the glass to form volatile SiF_4_, while ion bombardment provides physical sputtering and directional assistance, enabling anisotropic etching. Li et al. [45] reported the implementation of DRIE on Pyrex glass using SF_6_ plasma. At a low pressure of 0.2 Pa and a high self-bias voltage of −390 V, they achieved an etching rate of approximately 0.6 μm/min and a smooth surface with a roughness (Ra) of about 4 nm, resulting in through-hole structures with a bottom angle of approximately 88° and an aspect ratio greater than 10. These studies had shown that high-energy ions were crucial for removing non-volatile fluorides (such as NaF and AlF_3_) formed by elements like Na and Al in the glass, which directly determined whether the etching process could continue. The DRIE process is highly sensitive to the mask aperture size, and the vertical etching profiles, high aspect ratios, and the through-etching of 200 μm thick Pyrex glass can only be achieved when the mask opening is narrower than 20 μm [45]. For larger apertures, the increased exposure area exacerbates the deposition of non-volatile products on the sidewalls, causing the holes to degenerate into sloped or V-shaped profiles.

In addition to directly fabricating micropores on glass substrates, the DRIE process can also be employed to create a mold with a shape opposite to the target feature, followed by glass reflow to form microstructures or micropores. Kuang et al. [46] derived theoretical equations for the glass reflow process based on an analogy between fluid dynamics and circuit equations and analyzed the effects of parameters such as heating time, temperature, gap width, and surface tension on the reflow rate. Both simulation and experimental results indicated that the theoretical curve of glass reflow length over time aligned with the trend of experimental data and is largely consistent numerically. The theoretical model provides a basis for predicting the filling behavior of glass within microchambers and can guide the optimization of process parameters to avoid cavity defects caused by incomplete reflow. However, the industrial feasibility of plasma etching TGVs remains limited by processing costs and production capacity constraints and is primarily confined to research environments and high-performance application areas. The glass reflow process is limited by capillary filling limits; microchambers with high aspect ratios may not fill completely due to insufficient driving force.

### 2.6. Others

Wet etching is another important technical route for preparing TGVs. Unlike dry etching, it does not require an external electric field or plasma assistance, and mainly relies on the chemical reaction between hydrofluoric acid (HF)-based etching solution and silicon dioxide (SiO_2_) in the glass to achieve selective material dissolution [47,48]. The process is essentially isotropic; while the etchant corrodes vertically, it also laterally etches the glass beneath the mask, causing the sidewalls of the through-holes to typically exhibit a certain taper [49]. To balance etching rate and uniformity, it is necessary to maintain a stable H^+^ concentration in practical processes; regarding masks, shallow etching often uses photoresists such as SU-8 or AZ4620, while for deep through-holes, corrosion-resistant metal masks such as Cr/Au against HF are preferred. In the wet etching route, using photo-sensitive glass for selective etching is a rather distinctive process path. Wang et al. [50] successfully fabricated glass through-holes on photosensitive glass with a depth-to-width ratio of approximately 8:1 and a minimum diameter of 25.68 μm by combining ultraviolet exposure, thermal treatment, and wet etching methods. The study showed that the through-hole diameter increased with the exposure dose. This process fully exploits the material selectivity advantage of wet etching and is suitable for the mass production of through-hole interposer boards. However, for ordinary borosilicate glass that does not possess photosensitive properties, through-holes can also be fabricated by directly using high-concentration HF for wet etching, but it is necessary to overcome the challenge of maintaining mask integrity during prolonged etching. Ding et al. [51] applied a 49% HF solution and employed Cr/Au enhanced with dehydrated AZ4620 photoresist as a mask to achieve through-etching of 500 μm thickness on borosilicate glass. Due to the inherent isotropy of wet etching, the lateral etching rate was approximately twice that of the vertical etching rate. The final through-hole sidewall had an inclination angle between 45° and 50°. Sahu et al. [52] utilized a Cr film combined with an AZ1512HS positive photoresist as the mask layer and etched Borofloat glass in a 20% HF solution for 300 min, achieving the preparation of through-holes with a depth of 500 μm. These studies indicate that by optimizing the mask structure and the etchant concentration, it is also possible to directly etch through-hole structures on ordinary glass at a relatively high etching rate, but their sidewall verticality and depth-to-width ratio are fundamentally limited by the isotropic etching characteristics. Therefore, the wet etching approach is more suitable for applications where the verticality requirements of the sidewalls are relatively relaxed, while it still faces challenges in the preparation of high-performance TGVs that require extremely high aspect ratios and steep sidewalls.

### 2.7. Summary

Table 2 summarizes the key performance metrics for the non-laser methods in terms of hole quality, metallization uniformity, and thermomechanical reliability. In terms of hole quality, sandblasting and mechanical drilling exhibit diameter deviations of tens of micrometers, with aspect ratios typically below 5:1 [11,21,22,23,24]. In contrast, the photosensitive glass method and DRIE achieve aspect ratios of >35:1 and >10, respectively, while sidewall roughness decreases from the micrometer scale of sandblasting to the nanometer scale of DRIE [45,46]. Although ECDM can process glass of any thickness, the heat-affected zone (48–87 μm) and radial overcutting (73–160 μm) severely limit hole profile accuracy [37,41,42,43,44]. Regarding metallization uniformity, the shadowing effect caused by rough pore walls easily leads to discontinuities in the seed layer and voids in the fill, whereas photoresist glass (Ra < 1 μm) and DRIE (Ra ~ 4 nm) provide superior substrates for metallization [29,30,31,32,33,34,45,46]. The core vulnerability of thermal–mechanical reliability lies in microcracks and subsurface damage introduced during processing. Surface defects from sandblasting, exit chipping from mechanical drilling, and cracks in the heat-affected zone from ECDM can all propagate into interfacial delamination failure during thermal cycling between −40 °C and +125 °C [37,41,42,43,44]. While non-laser methods laid the foundation for the TGV technique and remain valuable for large-sized structural holes and prototype devices, their overall performance in terms of hole quality, metallization, and reliability is no longer sufficient to meet the requirements of advanced packaging for fine pitch, high aspect ratios, and long-term durability.

## 3. Laser Machining for TGVs

With the advancement of ultrafast laser techniques and the industry’s urgent need for high-precision, high-throughput TGV fabrication, laser-based special processing methods have become the mainstream approach in its manufacturing. Compared to non-laser methods, laser-based techniques have achieved significant improvements in hole diameter control, aspect ratio capabilities, processing speed, and processing quality. Thereafter, these techniques have gradually transitioned from laboratory settings to large-scale industrial production.

### 3.1. Laser Ablation Drilling

**Figure 6:**
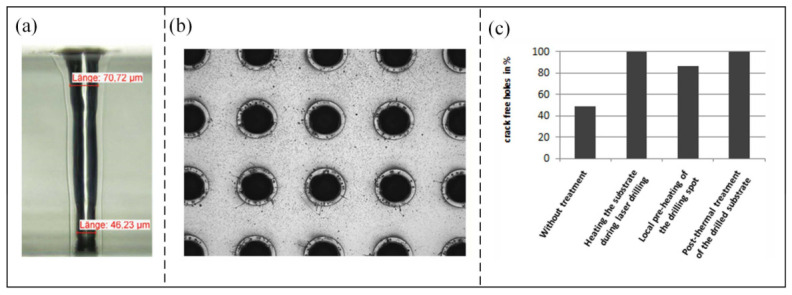
Morphology, array characteristics, and crack-resistance reliability of glass TGVs fabricated using two typical CO_2_ laser processes [57]: (**a**) cross-sectional morphology of a nearly cylindrical through-hole with a diameter of less than 100 μm; (**b**) uniform through-hole array with a pitch of 400 μm; (**c**) statistical comparison of crack-free hole ratios after 500 thermal cycles from −55 °C to 125 °C under different thermal pre- and post-treatment conditions in 500 μm thick Schott D263Teco glass.

Laser ablation drilling (LAD) is the most straightforward method of laser hole formation and is one of the first techniques attempted in TGV laser processing. The principle involves utilizing the high peak power density of pulsed lasers (typically in the range of 10^8^ to 10^12^ W/cm^2^) to instantly heat the glass material at the laser focal point to temperatures above the vaporization point. The material is then removed layer by layer in the form of vapor and molten ejecta, ultimately forming a through-hole. In TGV processing, commonly employed laser sources include nanosecond UV lasers (such as 355 nm Nd: YAG third-harmonic generation), CO_2_ lasers (10.6 μm wavelength), and ultrashort-pulse lasers (picosecond and femtosecond), which have widespread application in recent years [53,54,55]. Nanosecond UV lasers are highly favored due to their high photon energy and effective absorption by most glass materials. By controlling the laser beam trajectory via a galvanometer scanning system, various processing modes such as trepanning or percussion drilling can be achieved. Chung and Lin [56] performed “crack-free” through-hole drilling (hole diameter approximately 200–300 μm) on Pyrex 7740 glass using a CO_2_ laser by controlling heating and cooling gradients. Under optimized parameters, a through-hole was etched within seconds at a power of 6 W and an etching rate of 11.4 μm/s, with no significant macroscopic cracks on the sidewalls. This study demonstrates that CO_2_ lasers can achieve macroscopically crack-free glass through-holes through thermal stress management. However, the evolution of residual thermal stress during long-term thermal cycling still warrants attention. Brusberg et al. [57] conducted CO_2_ laser drilling on 500 μm thick glass substrates, demonstrating the ability to fabricate arrays of through-holes with diameters < 100 μm. The processing time per hole was reduced to hundreds of milliseconds, and heating pre-treatment and post-treatment significantly reduced hole cracking, as shown in Figure 6. However, CO_2_ laser drilling has a narrow process window regarding hole quality (taper, roughness), and tapered profiles and high roughness directly threaten the continuous coverage of the seed layer, affecting the uniformity of subsequent metallization processes. Chung et al. [58] implemented a poly dimethyl siloxane (PDMS) protective layer process to assist CO_2_ laser processing. Experimental results showed that the microchannel width decreased from 150 μm in air to 137 μm (with a 150 μm thick PDMS protective layer). Simulations combined with experiments further demonstrated that the protective layer enabled a smaller temperature gradient, thereby reducing the heat-affected zone and minimizing crack formation. However, the removal and residue issues associated with the protective layer pose new challenges for the cleanliness requirements of subsequent metallization. For glass interlayer applications, CO_2_ lasers have successfully drilled holes with diameters less than 100 μm in glass 145–500 μm thick [59]. Although a seed layer is deposited via sputtering followed by copper electroplating, hole wall roughness and taper remain bottlenecks for mass production.

**Figure 7:**
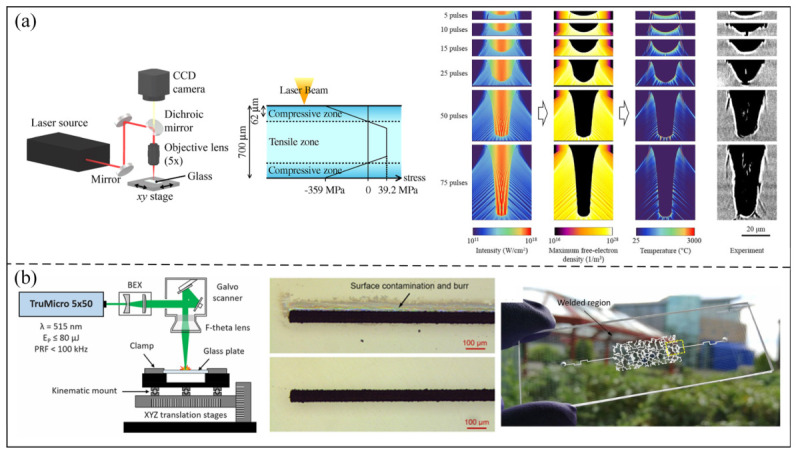
Femtosecond laser drilling damage mechanisms and picosecond laser fabrication of glass microfluidic devices. (**a**) Left, schematic of the femtosecond laser experimental setup; middle, calculated stress distribution around the laser-irradiated region in chemically strengthened glass; right, evolution of optical intensity, free-electron density, temperature distribution, and experimental hole morphology under 5–75 laser pulses [62]. (**b**) Left, schematic of the picosecond laser micromachining setup; middle, glass microchannel morphologies before and after cleaning; right, fabricated Berea sandstone-like glass microfluidic device and its welded region [63].

The cold processing characteristics of femtosecond lasers significantly outperform those of long-pulse lasers in terms of hole quality (microcrack suppression and extremely narrow HAZ), providing an important technical approach for high-precision TGV fabrication. Huang et al. [60] utilized a femtosecond fiber laser to fabricate micro holes with diameters < 50 μm in glass, achieving an aspect ratio of 8:1, an extremely narrow heat-affected zone (<5 μm), virtually no remelted layer on the hole walls, and smooth cutting edges. The extremely low thermal damage is beneficial for thermomechanical reliability, and the narrow HAZ also reduces the risk of crack propagation during subsequent thermal cycling. Sakakura et al. [61] quantitatively observed the propagation of stress waves and the distribution of thermal stress fields during femtosecond laser machining using pump-probe imaging techniques. Experimental data revealed that under longer pulse durations, both the threshold for stress wave generation and the sensitivity of stress wave amplitude to changes in pulse energy were higher. Even in the absence of visible cracks on the hole walls, residual stresses may drive crack initiation or interfacial delamination during thermal cycling, directly contributing to reliability failure mechanisms. Ito et al. [62] employed high-speed imaging, combined with numerical analysis of propagating stress waves and temperature distributions, as shown in Figure 7a. They found that damage to the hole walls and bottom was primarily caused by stress waves, while damage around the hole aperture was mainly due to thermal stress relaxation. This study reveals the temporal evolution of stress propagation in the material and the temperature distribution generated by excited electrons. Wlodarczyk et al. [63] utilized picosecond lasers to rapidly fabricate microfluidic channels and through-holes in glass without a mask, as shown in Figure 7b. Experimental results demonstrated that at a glass thickness of 250 μm, processing speeds could reach >10 mm/s.

The primary advantage of LAD lies in the directness of its processing principle and the relative maturity of the equipment system. This is because through-hole fabrication can be completed in a single step without post-processing. However, this advantage comes at the expense of processing quality. The intense thermal effects generated by high-energy laser pulses in glass lead to the formation of remelted layers and microcracks on the hole walls, as well as chipping at the hole edges and the creation of a heat-affected zone. More seriously, the conical nature of the laser beam and the exponential decay of energy with depth result in through-holes that often exhibit a pronounced conical profile (wider at the entrance and narrower at the exit), which affects the uniformity of subsequent metallization.

### 3.2. Laser-Induced Deep Etching

Laser-induced deep etching (LIDE) is currently the most influential and commercially promising technical solution in the field of TGV manufacturing [64,65,66]. This technique was first commercialized by the German company LPKF, and its core concept involves combining ultrafast laser modification with wet chemical etching. The process begins by applying a picosecond laser to a glass substrate to form an induced zone with a diameter of approximately 1 micrometer. The glass is then immersed in hydrofluoric acid or an alkaline solution; the etching rate in the laser-induced region is significantly higher than in other areas of the glass, causing this region to expand and form a through-hole [64].

**Figure 8:**
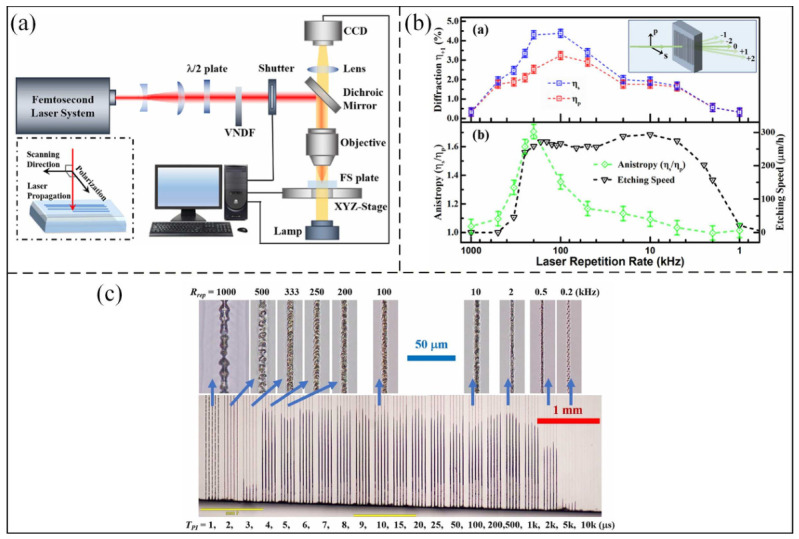
Mechanisms and applications of femtosecond laser-assisted chemical etching of glass; (**a**) FLISE setup; (**b**) grating anisotropy vs. etching speed (**c**) etched microchannels (0.2–1000 kHz, 2.0 μJ, 1 mm/s) [65].

LIDE is suitable for processing blind holes or arrays of any size and pitch. It can fabricate holes of any shape or larger dimensions by connecting closely spaced through-holes (1–10 μm) [67]. Ostholt et al. [68] demonstrated the feasibility of high-quality, high-precision deep microstructural processing of glass using a two-step LIDE process (integrating rapid laser modification and chemical etching). Experimental results demonstrated that for glass with thicknesses ranging from 50 to 200 micrometers, TGV diameters reached 10 to 50 μm with a taper angle of less than 5°. As an early study of LIDE, this provides a foundational reference for understanding the fundamental framework and industrialization potential of the LIDE process. In the LIDE process, precise control of the glass damage threshold is crucial for process optimization and scaling up production. The laser energy input must exceed the modification threshold to induce sufficient etching selectivity, yet it must not exceed the damage threshold to prevent microcrack formation. Vanda et al. [69] proposed using laser damage threshold (LIDT) testing as a tool to optimize the process window for TGV processing of D263 glass wafers. They measured the LIDT of D263 glass at two wavelengths, 1030 and 515 nm, and utilized these data to establish upper limits for the process parameters of a multi-Bessel beam processing optical system. Qi et al. [65] examined the effects of laser pulse energy, repetition rate, and writing speed on the selective etching rate in fused silica using a 1030 nm femtosecond laser system, as shown in Figure 8. The experiments revealed that the initiation of FLISE required a single-pulse energy exceeding a certain threshold and that there was an operational window for the laser repetition rate between upper and lower thresholds, enabling a constant etching rate of approximately 280 (±30) μm/h for the fabrication of microfluidic devices. The upper limit was constrained by thermal diffusion, while the lower limit was determined by whether the modified region could be fully penetrated. Serkov and Snelling [66] demonstrated that spatially resolved femtosecond laser irradiation could induce controllable enhancement of chemical reactivity in borosilicate glass at irradiance levels below the damage threshold. They found that the HF etching rate was closely correlated with a decrease in the optical transmittance of the glass at 488 nm, and that this change in transmittance was regulated by the generation of boron-oxygen hole centers. Consequently, laser irradiation combined with chemical etching yields lower surface roughness than either laser or chemical etching alone. Processing below the damage threshold prevents microcracks in the hole walls, fundamentally improving thermal cycling reliability, and the low surface roughness of the hole walls facilitates uniform coverage of the subsequent metallization seed layer.

The LIDE process completely avoids the formation of microcracks and thermal damage throughout the entire fabrication process. This is because energy deposition during the laser modification stage is strictly confined to the focal volume, preventing melting of the surrounding material or the generation of thermal stress [70,71]. The etching stage is a mild chemical process that introduces no mechanical stress [72]. However, LIDE still faces several challenges. First, the two-step process increases production complexity compared to a one-step method, requiring additional chemical processing facilities and wastewater treatment systems. Second, the etching process is inherently isotropic, meaning that while vertical etching occurs, some lateral etching also takes place, leading to pore enlargement and limitations on the aspect ratio. Furthermore, different types and thicknesses of glass respond differently to laser wavelengths and etching solutions, and the transferability of process parameters needs to be improved.

### 3.3. Femtosecond Laser-Assisted Etching

**Figure 9:**
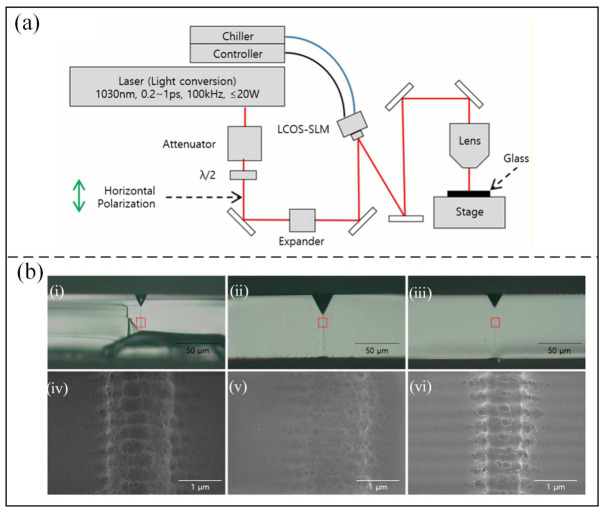
Process and performance of laser-induced selective etching for TGV fabrication: (**a**) Schematic of spatial light modulator-based Bessel beam laser modification system. (**b**) Cross-sectional characterizations of laser-modified regions: (**i**,**iv**) single pulse, (**ii**,**v**) 213 ps double pulses, and (**iii**,**vi**) 10 ns double pulses; (**i**–**iii**) optical micrographs and (**iv**–**vi**) nanograting SEM images [76].

Femtosecond laser-assisted etching (FLAE) is an important variant of LIDE and one of the most actively researched methods for TGV fabrication [73,74]. While it shares the same core concept as LIDE (etching following laser modification), it differs in specific implementation details. In FLAE, a femtosecond laser pulse is tightly focused and scanned along a pre-programmed three-dimensional trajectory across a glass substrate to locally modify the material. Subsequently, the modified regions are selectively removed via chemical etching (using KOH or HF solutions) to form through-holes. Chen and Yu [75] utilized picosecond laser-induced selective etching to fabricate TGVs on borosilicate glass. They found that a series of nanoscale cavities formed along the beam path within the laser-affected zone (LAZ), which was the key microscopic mechanism for enhancing etching selectivity. Experimental data indicated that HF concentration and glass composition had a more significant impact on the TGV profile than laser pulse parameters. Under the optimized conditions, vertical through-holes with near-90° angles were achieved. The nanoscale cavity mechanism plays a significant role in controlling hole quality (verticality) and facilitates uniform coverage of the subsequent PVD seed layer and cavity-free copper filling. Kim et al. [76] modified commercial glass using a Bessel beam combined with ultrashort dual pulses (spaced 213 ps and 10 ns apart), followed by etching with an 8 mol/L KOH solution (110 °C). They found that photonic absorption was more effective than thermal absorption, and the dual-pulse scheme helped increase TGV processing speed, as shown in Figure 9. This study directly validates the feasibility of fabricating TGVs via KOH etching after femtosecond laser modification using Bessel beams, providing new insights into process acceleration from the perspective of photonic absorption mechanisms. Kiyama et al. [74] compared the etching effects of KOH and HF on femtosecond laser-modified fused silica. They found that the etching selectivity of concentrated KOH aqueous solution was superior to that of HF, enabling the fabrication of microchannels with centimeter-scale lengths and diameters < 60 μm (aspect ratio approximately 200). Photoluminescence and confocal Raman spectroscopy indicated that Si-rich structures formed in the laser-modified region, which was the primary reason for the high selectivity of KOH etching. Additionally, there have been studies on vertical sidewalls [75], low-roughness surfaces [76,77], and uniform pore patterns [78] in TGV fabrication using LIDE techniques, which provide a foundation for high-quality seed layer coverage and uniform electroplating. Like LIDE, FLAE also faces a trade-off between processing speed and single-pore quality. Although parallel processing can be achieved through multi-beam splitting techniques to increase throughput, this also leads to increased system complexity and cost.

### 3.4. Others

In addition to the mainstream two-step method described above, several other TGV fabrication methods based on laser principles are worth noting. Among these, the quasi-continuous-wave (QCW) laser rapid drilling technique enables the rapid fabrication of TGVs in a single step by engineering the pulse width and repetition rate. Matsumoto et al. [77] proposed a high-speed TGV processing method based on QCW laser pulse trains, which enabled high-aspect-ratio through-holes in 50 μm and 100 μm thick glass within 10–20 μs using a single laser source, without the need for post-processing. Studies demonstrated that this method combined high-speed processing capabilities with high-aspect-ratio processing advantages and was compatible with high-speed galvanometer scanning systems, showing significant potential for industrial mass production. However, there is still room for further optimization regarding hole diameter consistency and hole shape uniformity. To further enhance TGV processing efficiency, laser beam shaping and parallel processing technologies have gradually garnered attention. Such methods typically utilize diffractive optical elements (DOEs) or spatial light modulators (SLMs) to modulate the laser wavefront, splitting a single laser beam into a multi-focus array to enable simultaneous multi-hole processing. Yoshizaki et al. [78] demonstrated an SLM-based parallel transient selective laser processing method capable of simultaneously machining two holes, each approximately 100 μm deep, within about 50 μs, significantly improving processing efficiency and throughput. However, this method imposes stringent requirements on optical modulation precision and focal energy distribution, and it still faces challenges regarding processing stability and repeatability under large-scale parallel processing conditions. Furthermore, the volume laser-induced structuring (VLIS) process, jointly developed by Plan Optik and 4JET, is also based on laser-induced volume modification and wet etching and has been optimized in terms of dynamic beam focusing, path accuracy control, and metallization process integration. Related results indicate that the VLIS process enables high-precision, high-consistency batch manufacturing of TGVs, with inter-hole positioning accuracy better than ±2 μm and features such as the absence of microcracks and smooth hole walls. It demonstrates significant application potential in large-scale production scenarios such as advanced packaging and display substrates [79,80].

### 3.5. Summary

Table 3 summarizes the key performance metrics of the laser TGV processing methods across three dimensions: hole quality, metallization uniformity, and thermomechanical reliability. In terms of hole quality, CO_2_ laser ablation can produce through-holes with diameters < 100 μm and no macroscopic cracks, but issues with taper and recast layers remain prominent [56,57,58,59]; femtosecond/picosecond laser ablation, leveraging its “cold processing” characteristics, compresses the heat-affected zone to <5 μm and increases the aspect ratio to over 8:1, significantly suppressing microcrack formation [60,61,62,63]. As representative two-step methods, LIDE and FLAE have achieved a qualitative leap in hole quality, with aspect ratios as high as 200:1, sidewall perpendicularity approaching 90°, and surface roughness superior to that of single-laser or chemical etching [66,67,68,69,75,76]. QCW lasers, with a single-hole formation time of 10–20 μs and a high aspect ratio, balance both hole quality and production capacity [77]. Regarding metallization uniformity, the tapered hole walls and rough surfaces produced by CO_2_ laser ablation severely hinder the continuous coverage of the seed layer [57,59]. In contrast, the vertical sidewalls and low-roughness surfaces of LIDE/FLAE provide an ideal substrate for the uniform deposition of the PVD seed layer and void-free copper filling [68,69,75]. Regarding thermomechanical reliability, the residual stresses and microcracks introduced by laser ablation are the primary causes of thermal cycling delamination failure [61,62]. In stark contrast, the LIDE/FLAE process fundamentally avoids thermal damage and microcrack formation through modification mechanisms operating below the damage threshold, offering an inherent advantage in thermomechanical reliability [67,69]. In summary, laser processing methods have surpassed non-laser methods across three dimensions: hole quality, metallization uniformity, and thermomechanical reliability. Among these, two-step methods represented by LIDE and FLAE have become the mainstream technological approach for TGV manufacturing due to their damage-free nature, high precision, and excellent process controllability. Also, breakthroughs in QCW lasers and parallel processing techniques have provided a viable path for increasing production capacity for large-scale mass production.

## 4. Application of TGVs

Owing to the exceptional electrical, thermal, and mechanical properties of glass substrates, the TGV technique has found widespread application across numerous high-tech fields, ranging from RF communications and high-performance computing to MEMS sensors and optoelectronic integration.

### 4.1. 2.5D/3D Packaging Interconnects

**Figure 10:**
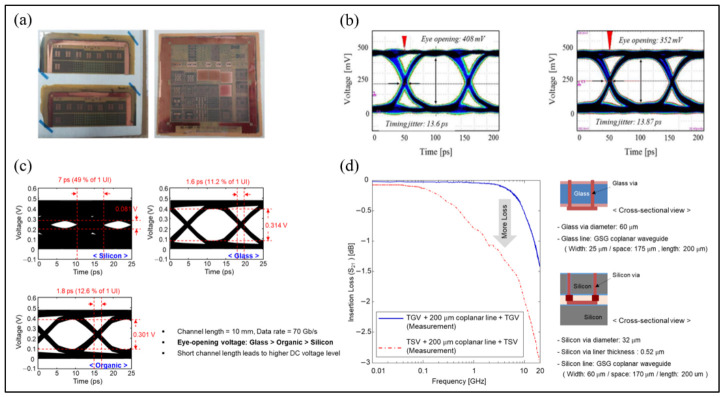
High-speed interconnection performance of glass interposers [81]: (**a**) fabricated glass interposer test vehicles; (**b**) simulated eye diagrams of glass, silicon, and organic interposer channels at 70 Gb/s; (**c**) measured insertion losses of TGV and TSV channels; (**d**) measured eye diagrams of TGV and TSV channels.

The primary application of TGVs is in glass interposers for 2.5D/3D packaging, where they provide vertical electrical interconnections between chips and between chips and the packaging substrate for high-density signal routing and power distribution. Glass interposers offer low dielectric loss in the GHz range, excellent dimensional stability, and a coefficient of thermal expansion closely matched to silicon chips, thereby reducing thermomechanical stress during thermal cycling. The high-speed interconnection performance of glass, silicon, and organic interposers was comparatively evaluated using fabricated test vehicles, electromagnetic simulations, and electrical measurements [81], as shown in Figure 10. The fabricated glass interposer coupons incorporated various TGV-based structures for signal-integrity and power-integrity characterization. For 10 mm long channels operating at 70 Gb/s, the simulated eye diagrams showed that the glass interposer provided the largest eye-opening voltage and the lowest timing jitter among the three substrate types. Measurements further demonstrated that the TGV channel exhibited lower insertion loss than the TSV channel over the GHz frequency range. At 10 Gb/s, the TGV channel achieved an eye-opening voltage of 408 mV and a timing jitter of 13.6 ps, compared with 352 mV and 13.87 ps for the TSV channel, respectively. These results demonstrate the advantages of glass interposers in maintaining signal integrity and supporting high-bandwidth, high-density 2.5D/3D integration for CPUs, GPUs, AI accelerators, and HBM.

### 4.2. MEMS Device Packaging

**Figure 11:**
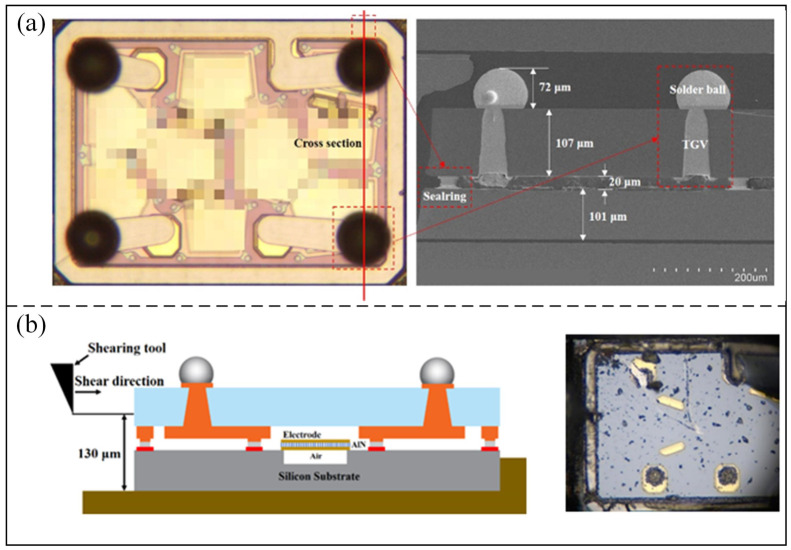
Applications of MEMS device packaging [84]; (**a**) The RF filter package with TGVs after solder ball formation; (**b**) The scheme and fracture section of the die shear test scheme.

MEMS packaging demands high hermeticity, mechanical reliability, and interconnect density, for which glass substrates are ideal candidates. In wafer-level MEMS packaging, TGV enables vertical interconnects integrated into glass-capping wafers, replacing wire bonding and reducing package size [82,83]. In a 3D wafer-level hermetic packaging solution, a glass interlayer with copper-filled TGVs served as the capping wafer for an RF filter. Combined with Au Sn TLP bonding, this approach achieved a shear strength of 54.5 MPa, far exceeding the standard requirement of approximately 6 MPa, with insertion loss variation below 0.2 dB after accelerated stress testing [84], as shown in Figure 11. This package passed consumer electronics reliability tests, meeting the demands of 5G high-frequency communication systems. In inertial sensors such as accelerometers and gyroscopes, the high CTE match between glass and silicon minimizes interfacial stress at the anodic bonding interface, ensuring long-term zero-bias stability. TGV-based vertical lead-out electrodes simplify packaging interconnect design and improve reliability. Pressure sensors find important applications in aerospace and atmospheric pressure monitoring, often employing vacuum-sealed structures such as micro resonators whose quality factor degrades with increasing ambient pressure due to air damping. The feasibility of TGV in capacitive pressure sensors has been demonstrated [85].

### 4.3. Optoelectronic Integration and Co-Packaged Optics

As data center traffic grows exponentially, interconnect bandwidth is advancing toward 1.6 Tbps and beyond. CPO, a key technique to break through the bandwidth bottleneck of traditional pluggable optical modules, is emerging as a compelling new application for TGVs [86,87]. The inherent advantages of glass substrates include optical transparency for direct laser alignment, simplifying fiber-to-silicon photonics chip coupling [88]. The dual properties of transparency and low loss enable coplanar integration of electrical and optical signals on a single glass platform via TGV and surface wiring. This approach reduces conversion losses and maintains alignment accuracy owing to CTE matching between glass and silicon.

**Figure 12:**
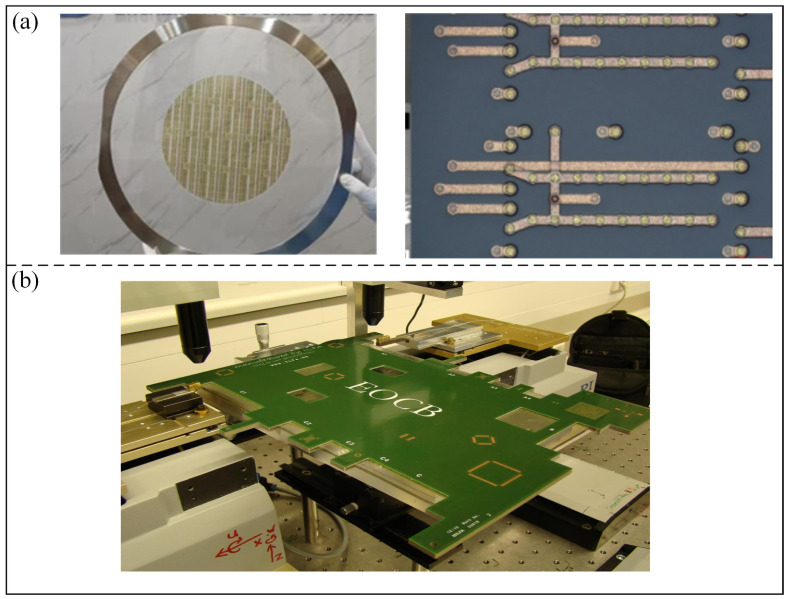
Applications of optoelectronic integration and CPO; (**a**) pioneering 110 GHz 8-inch wafer-level TGV interposer manufactured by SPVTECH [89]; (**b**) Fabricated multilayer electro-optical circuit board with embedded glass waveguide panels during optical characterization [90].

Recently, Shenzhen Photonics Valley Technology (SPVTECH), in collaboration with Shanghai Jiao Tong University and Shenzhen University, successfully developed China’s first 8-inch wafer-level TGV optoelectronic interlayer process, achieving a bandwidth of 110 GHz, as shown in Figure 12a. This interlayer uses femtosecond laser LIDE combined with multilayer RDL to achieve a 4:1 aspect ratio on a 230 μm thick glass substrate, supporting 2.5D/3D CPO integration of VCSELs, DMLs, EMLs, silicon photonics, and lithium niobate modules [89]. As shown in Figure 12b, multilayer glass waveguide panels can be embedded within electro-optical circuit boards to provide high-density optical routing between on-board photonic modules and external fiber-array interfaces [90].

### 4.4. Other Emerging Applications

TGVs also show promise in several emerging fields. In Micro-LED displays, glass substrates provide high flatness, optical transparency, and tunable CTE, while TGVs construct high-density vertical interconnects between driving chips and display pixels, reducing parasitic resistance and signal delay. Garner et al. [91] integrated metallized TGV interconnects into TFT active matrix backplanes, verifying the feasibility of frameless Micro-LED tiled displays. In biomedical and implantable electronics, glass offers excellent biocompatibility, chemical inertness, and long-term stability [8]. At the same time, TGVs support 3D integration of microfluidic chips, biosensors, and signal processing circuits, reducing package size while improving integration. The optical transparency of TGV substrates also suits optogenetic, bioimaging, and wearable medical systems requiring photoelectric collaboration. In quantum computing and quantum information systems, which demand low dielectric loss, low thermal conductivity coupling, and high signal integrity in cryogenic environments, glass materials offer lower microwave transmission loss and better insulation than silicon or organics.

Fraunhofer IZM and University of Mainz developed a glass electro-optical chip carrier for ion trap quantum processors at 532 nm, using TGV for electrical connection and heat dissipation, with metallized surfaces supporting welding and lead bonding [92]. Although still exploratory, TGV’s low-loss microwave transmission and integrated optical functions make it a promising route for quantum devices. For high-frequency automotive electronics, glass substrates feature low dielectric constant, low dielectric loss, and excellent dimensional stability, effectively reducing millimeter wave signal loss and distortion [93]. Su et al. [94] reported a high-bandwidth compact antenna packaging scheme for 77 GHz automobile radar using five-layer glass stacking with TGV interconnection. The package size was 10 × 9 × 1 mm^3^ with gain exceeding 7 dB, achieving direct interlayer signal transmission and coupling through TGV and RDL.

## 5. Discussion

The previous sections provide a systematic overview of the key performance characteristics of TGV processing methods and their typical application scenarios. This section delves into an in-depth discussion from three perspectives: key technical challenges, trade-offs among the various machining methods, and the current state of reliability research, with the aim of revealing the underlying logic and bottlenecks in the current development of TGV techniques.

### 5.1. Core Challenges in Hole Quality

The ultimate performance of TGVs depends largely on the geometric quality and structural integrity of the through-holes, both of which are influenced by a combination of manufacturing methods, process parameters, and the properties of the glass material. Controlling the hole geometry is the most fundamental challenge in TGV manufacturing. An ideal TGV should have a cylindrical profile with vertical sidewalls and a smooth surface to facilitate the continuity of subsequent seed layer deposition, the uniformity of electroplating fill, and impedance matching for high-frequency signals [95,96]. However, in actual processing, through-holes often exhibit non-ideal profiles such as hourglass, conical, or V shapes. Due to their inverted trapezoidal geometry, the waist region of hourglass-shaped TGVs imposes stringent requirements on step coverage during PVD sputtering. For instance, the bottom and areas below the waist require substrate flipping to ensure full coverage of the seed layer. Key dimensional control of the hole profile, including the proportional relationships between the top diameter, bottom diameter, and waist diameter, directly affects the sidewall angle and the feasibility of subsequent filling processes [31,97,98].

Microcracks and structural damage present unique challenges for glass as a brittle material [99,100]. Regardless of the processing method, crack initiation and propagation in glass substrates remain the primary threat to TGV reliability [9]. For example, in laser processing, excessively high pulse energy or insufficient cooling could generate residual thermal stress in the glass, leading to radial cracks and debris on the hole walls. In wet etching processes, if the etching selectivity ratio between the modified and unmodified regions is insufficient, the isotropic corrosion of the etchant may cause pitting and stress concentrations at the hole edges. Thereafter, optimizing the laser pulse energy density to be just above the glass modification threshold and well below the damage threshold is key to suppressing cracks.

Sidewall roughness significantly affects the high-frequency electrical performance of TGVs [101,102]. In the millimeter-wave and higher-frequency bands, the skin effect causes the current to concentrate in an extremely thin layer on the conductor surface; the increased surface area and localized scattering resulting from sidewall roughness significantly increase conductor losses. Advanced processes such as LIDE can control sidewall roughness to an excellent level of Ra ≤ 0.08 μm [103], but maintaining this quality under high-throughput conditions remains a challenge.

### 5.2. Effects of Glass Composition on Process Selection and via Quality

The selection of TGV fabrication methods is closely related to the composition and properties of glass materials. Different glass substrates, such as borosilicate glass, fused silica, D263 glass, and photosensitive glass, differ in thermal expansion, optical absorption, chemical etching response, mechanical brittleness, and process compatibility. These differences influence the choice of machining route, achievable via profile, sidewall quality, and reliability after metallization. Therefore, glass composition should not be considered only as a substrate parameter but also as an important factor affecting process selection and interconnect stability. A brief comparison of the advantages, limitations, and suitable TGV methods for typical glass materials is summarized in Table 4.

For borosilicate glass and D263 glass, their relatively good processability makes them suitable for laser drilling, LIDE, and wet-processing-assisted TGV fabrication, but laser energy and post-etching conditions must be controlled to reduce thermal stress and microcrack formation. Fused silica has excellent thermal stability and low dielectric loss, making it attractive for high-frequency applications; however, its high hardness and high chemical resistance increase the difficulty of efficient via formation. Photosensitive glass provides a unique material route for selective exposure, crystallization, and etching, which is beneficial for forming smooth and precise vias, but its application is restricted by material availability and process complexity.

### 5.3. Comprehensive Comparison of Machining Methods

The selection of TGV machining methods involves trade-offs across multiple dimensions: quality, speed, cost, and scalability. Currently, no single method holds an absolute advantage in all dimensions. In terms of quality, LIDE and its variants (FLAE) lead the way due to their microcrack-free, smooth sidewalls, followed closely by the photosensitive glass method (which offers high precision but is limited by material constraints). DRIE offers the best sidewall verticality, but capacity and cost issues make it difficult to scale up. In terms of speed, the QCL laser method achieves single-hole processing at 10–20 μs per hole [77], while LPKF’s LIDE system has achieved a mass production speed of 5000 holes per second [104]. Traditional methods such as ECDM and DRIE lag significantly behind in terms of speed. In terms of cost, sandblasting and mechanical drilling require the lowest equipment investment but are limited in quality. Although laser-based methods involve higher equipment investment (a femtosecond laser TGV system typically costs hundreds of thousands to millions of dollars), their high throughput and the ability to spread costs across panel-level processing make the manufacturing cost per through-hole competitive in mass production. Scalability is another key factor that must be considered for the industrialization of TGV. Panel-level processing places higher demands on the travel range of laser scanning systems, the uniformity of etch channels, and overall panel stress management. Some companies have established high-tech wet-processing demonstration lines for large-panel TGV substrates, capable of consistently fabricating high-density TGVs on large-size panels [105].

To facilitate a more quantitative and direct comparison, the key performance indicators including achievable via diameter, aspect ratio, sidewall quality, throughput, process cost, and industrial scalability for representative TGV fabrication methods (sandblasting, mechanical drilling, DRIE, wet etching, ECDM, CO_2_ laser drilling, fs/ps laser ablation, LIDE/SLE, and QCW laser drilling) are summarized in Table 5. Specifically, LIDE demonstrates competitive performance with near vertical, crack-free vias (diameter ~ 10–60 μm), 8-inch wafer-level demonstration, and 110 GHz bandwidth capability [68,75,76,81,105], whereas competing techniques such as CO_2_ laser drilling suffer from HAZ and cracking [56,57,58,59], DRIE offers excellent precision but suffers from low throughput (~0.6 μm/min) and high equipment cost [45,46], mechanical drilling is suitable for large vias but limited by tool wear and serial processing [25,26,27,28], and ECDM provides low cost but is limited by HAZ, overcut, and taper [37,42,43,44].

### 5.4. Influence of TGV Fabrication Methods on Metallization and Copper Filling

In practical TGV manufacturing, hole formation is only the first step, while seed layer deposition, barrier/adhesion layer preparation, copper filling, and planarization directly determine whether the through-hole structure can be converted into a reliable vertical interconnect. For high-aspect-ratio TGVs, continuous and conformal seed layer coverage is difficult to achieve, especially when PVD sputtering is used, because non-ideal via profiles may cause shadowing effects at the lower sidewalls, via bottom, or waist region of hourglass-shaped holes. Once the seed layer becomes discontinuous or locally thin, the current distribution during electroplating becomes non-uniform, increasing the possibility of incomplete filling, seam formation, void generation, and local resistance increase. Therefore, via profile, sidewall roughness, taper angle, and microcrack distribution should be considered not only as indicators of hole quality but also as key factors affecting subsequent metallization and copper filling reliability [106,107].

Different TGV fabrication routes show different compatibility with metallization and copper filling. LIDE, FLAE, and photosensitive-glass-based selective etching methods generally produce smoother sidewalls and fewer microcracks, which are favorable for seed layer continuity, interfacial adhesion, and uniform Cu deposition [108]. DRIE provides good sidewall verticality and dimensional control, but its low throughput and high cost restrict its large-scale application in glass substrates. In contrast, mechanical drilling and sandblasting are low-cost methods, but edge chipping, subsurface damage, and rough sidewalls may reduce metal adhesion and cause local electric-field concentration during electroplating. Thermal laser drilling methods, such as CO_2_ laser ablation and QCW laser drilling, have advantages in processing efficiency, but heat-affected zones, taper, recast layers, and microcracks may increase the difficulty of conformal metallization and void-free copper filling. Therefore, these methods usually require post-treatment, such as wet etching, surface smoothing, cleaning, activation, or optimized adhesion/barrier layers, before reliable metallization can be achieved.

Copper filling defects are closely related to both via morphology and seed layer quality. In high-aspect-ratio vias, restricted transport of plating additives and metal ions may cause copper to deposit preferentially near the via opening, leading to premature closure and internal voids. Trapped gas, insufficient wetting, rough sidewalls, and discontinuous seed layers can further promote non-uniform copper growth. These defects may not only increase the electrical resistance of TGV interconnects but also become weak points during thermal cycling and package assembly. Therefore, reliable TGV manufacturing requires the integration of low-damage via formation, surface cleaning, seed/barrier layer optimization, copper filling control, and package-level reliability design [109].

### 5.5. Reliability

On the basis of hole formation, metallization, and copper filling quality discussed above, the reliability of TGVs, particularly their thermomechanical reliability, depends largely on the combined quality of via preparation and interconnect formation. Different hole-drilling methods, such as laser ablation, LIDE, and FLAE, introduce defects of varying types and severity, which directly become the source of subsequent failures. For example, traditional laser ablation tends to generate microcracks and a remelted layer around the hole walls. Under thermal cycling, these microcracks can propagate, leading to cracking of the glass substrate. Conversely, if wet etching lacks sufficient selectivity, it can cause indentations on the hole walls or stress concentration at the hole edges. In contrast, LIDE and FLAE, which employ a two-step process combining laser modification and chemical etching, can produce smoother, crack-free hole walls [68,69,75,76]. However, if laser parameters are not properly controlled (e.g., excessively high-pulse energy or inappropriate scanning speed), areas with uneven local modification may remain, which subsequently become weak points under thermal stress.

From the perspective of failure modes, defects in the hole preparation method directly determine the weak points of interconnects. Interface delamination is common when hole walls are excessively smooth or contaminated. This is because smooth hole walls reduce the mechanical anchoring effect between copper and glass, while debris left by laser ablation affects the adhesion of the seed layer. For hourglass-shaped or conical holes formed by laser etching, the waist region is prone to poor sidewall coverage during subsequent PVD sputtering, resulting in a locally thinner copper layer. During thermal cycling, this area is susceptible to accelerated electromigration due to excessive current density. Glass crack propagation is directly related to microcracks on the pore walls. During femtosecond laser modification, if the pulse energy density just exceeds the damage threshold rather than the modification threshold, radial microcracks could form around the pores. These cracks gradually extend during thermal cycling and eventually penetrate the substrate.

Although the thermomechanical reliability of TGVs is strongly affected by hole preparation defects, quantitative data show that stable performance can be achieved when via morphology, metallization, and filling quality are well controlled. Cu-metallized glass interposers with 35 × 120 μm TGVs and 100 μm pitch maintained electrical continuity after 1000 thermal cycles from −40 °C to 125 °C, with no obvious glass cracking, TGV failure, or Cu/Ti/glass interfacial separation [110]. In TGV-based 3D stacking structures, the mean resistance of a single TGV increased only from 9.11 mΩ to 9.97 mΩ after thermal cycling [111]. For Cu through-package vias in ultra-thin glass interposers, daisy chains passed 1000 thermal cycles without resistance change and the latest data reached 3000 cycles, although three out of 20 chains showed RDL-line delamination [112]. In addition, reliability tests of copper through-package vias in bare glass interposers also showed that electrical failure was not observed after thermal cycling, although local copper delamination and glass cracking could still occur under certain conditions [113]. These results suggest that long-term reliability depends on the combined control of sidewall damage, seed layer continuity, Cu/glass adhesion, copper filling quality, and package-induced stress.

Several optimization strategies have been developed to address reliability issues caused by laser-based machining methods. Strictly controlling the laser pulse energy within the range between the glass modification threshold and the damage threshold is key to suppressing microcracks [67]. In addition, surfactant-assisted or ultrasonic-assisted etching can reduce sidewall unevenness caused by isotropic etching, while the aspect ratio and cross-sectional shape of the holes should be adjusted according to the fabrication method. From the perspective of long-term reliability after metallization and package assembly, low-damage routes such as LIDE/FLAE and photosensitive-glass-based selective etching are generally more favorable because they reduce initial sidewall defects before seed layer deposition and copper filling. In contrast, direct thermal laser drilling, mechanical drilling, and sandblasting require post-treatment, surface activation, optimized adhesion/barrier layers, and carefully controlled Cu filling to achieve comparable long-term reliability. However, systematic comparisons of reliability data across different processes are still limited, and the coupling relationship between high-frequency electrical performance and thermal cycling life remains unclear.

## 6. Outlooks

Based on the preceding review and discussion, it is foreseeable that non-laser and laser machining for TGV fabrication will undergo a critical transition from process validation to high-efficiency and high-quality manufacturing over the next five to ten years. The following subsections, therefore, focus on the future trends of TGV machining methods, rather than the general development of the TGV technique.

### 6.1. Development of Glass Materials Compatible with TGV Machining

Currently, the glass materials utilized in TGVs are mostly mature products directly adopted from the display panel industry, such as Corning Eagle XG^®^, and are not specifically designed for semiconductor packaging requirements. Looking ahead, the targeted development of packaging-grade glass substrates will be a key direction. For non-laser methods, such as sandblasting, mechanical drilling, wet etching, ECDM, and DRIE, the brittleness, hardness, chemical stability, and etching selectivity of glass directly affect via profile accuracy, sidewall roughness, edge chipping, and crack formation. For laser-based methods, including CO_2_ laser ablation, femtosecond/picosecond laser ablation, LIDE, FLAE, and QCW laser drilling, optical absorption, thermal expansion, and laser-induced modification behavior are closely related to thermal damage, taper formation, and sidewall quality. For example, introducing a microcrystalline phase into the glass matrix could enhance the substrate’s strength and fracture toughness while maintaining excellent electrical insulation and processability. Alternatively, incorporating photosensitive properties could enable more efficient processing routes. Additionally, TGV processing of ultra-thin glass (thickness < 50 μm) will become an active area of research to meet the demand for ultra-thin packaging in wearable devices, flexible electronics, and 3D heterogeneous integration [114,115]. However, the handling and stress management of ultra-thin glass will be more challenging than that of conventional-thickness glass.

### 6.2. Continuous Evolution of Non-Laser and Laser TGV Machining Processes

In terms of TGV formation, both non-laser and laser machining processes still have significant room for improvement in machining accuracy, sidewall quality, processing efficiency, and wafer-level consistency. For non-laser methods, mechanical drilling and sandblasting are relatively mature and cost-effective, but tool wear, edge chipping, surface damage, and limited profile control remain major challenges. Wet etching and photosensitive glass methods are suitable for batch processing, but isotropic etching, mask dependence, and material limitations restrict their broader application. ECDM and DRIE provide alternative routes for insulating brittle materials and high-precision microstructures, but low efficiency, discharge instability, and high equipment cost still need to be addressed. For laser-based methods, the main challenge is to improve throughput while suppressing heat-affected zones, taper, microcracks, and recast layers. CO_2_ laser and QCW laser drilling show advantages in processing efficiency and are promising for high-throughput fabrication, whereas femtosecond and picosecond laser ablation provide higher precision and reduced thermal damage but suffer from relatively low throughput and high equipment cost. LIDE and FLAE, which combine laser modification with subsequent selective etching, are expected to play an important role in high-aspect-ratio TGV fabrication. In the future, these can be extended to intelligent optimization of process parameters [116,117], which is expected to significantly shorten process development cycles and improve yield rates.

### 6.3. Design-Process Reliability Co-Optimization

As TGV machining techniques mature, the evaluation of different fabrication routes will gradually shift from simply pursuing extreme manufacturing precision to system-level co-optimization involving process quality and reliability. Future TGV designs will need to strike an optimal balance between electrical performance, thermomechanical reliability, manufacturability, and process-induced defects. Different non-laser and laser machining methods may introduce different defect characteristics, such as edge chipping, microcracks, sidewall roughness, taper, residual stress, and thermal damage. These defects can further influence subsequent metallization, via filling, insertion loss, characteristic impedance, stress distribution, and fatigue life. Therefore, through multi-physics simulation, including electromagnetic–thermal–mechanical coupling, a complete chain linking machining parameters, via morphology, defect features, and performance metrics could be established. This allows the influence of process deviations on final device performance to be predicted during the design phase, thereby achieving process-aware design and improving the manufacturability and long-term reliability of glass interposers.

### 6.4. Driving Force from Emerging Applications

In the post-Moore’s Law era, emerging areas such as AI chip packaging, 6G communications and terahertz techniques, quantum computing, and autonomous driving sensors will provide strong application traction for TGVs. For example, the demand for interconnect bandwidth in high-performance AI chips (such as GPUs and NPUs) is growing at an astonishing rate. The high routing density and low signal loss characteristics of TGV glass substrates make them an ideal platform for 2.5D/3D packaging of AI chips. However, these applications also impose stricter requirements on machining accuracy, via density, sidewall smoothness, via profile consistency, and defect control. High-frequency and terahertz applications require smoother sidewalls and more stable via geometries to reduce signal loss and impedance mismatch. Intel’s strategic focus on glass substrates clearly demonstrates the importance of this direction. Furthermore, TGVs have been proven viable at a 110 GHz bandwidth and are expected to maintain excellent signal integrity at even higher frequencies in the future. Therefore, future application demands will not only promote the expansion of TGV technology but also directly drive the development of low-damage, high-precision, and high-throughput non-laser and laser machining processes.

## 7. Conclusions

This review focuses on the non-laser and laser-based machining methods for TGV fabrication in terms of hole quality, metallization, reliability, throughput, cost, and scalability. Based on the reviewed studies, several conclusions can be drawn.

(1) Non-laser methods (sandblasting, mechanical drilling, photosensitive glass processing, ECDM, DRIE, wet etching) are simple or offer good profile control but suffer from taper, chipping, rough sidewalls, material limits, low efficiency, high cost, or poor scalability.
(2) Laser-based methods provide flexibility, maskless processing, and high throughput. CO_2_ laser and ultrafast ablation enable rapid drilling, while LIDE and femtosecond-laser-assisted etching improve sidewall quality and suppress cracking. Thermal damage, residual stress, modification uniformity, and etching selectivity remain critical challenges.
(3) Via geometry and sidewall condition determine seed layer coverage, copper filling uniformity, interfacial integrity, and current distribution. TGV performance must therefore be evaluated together with metallization, filling quality, and thermomechanical reliability, not solely by hole formation accuracy.
(4) Future progress requires joint advances in glass materials, ultrathin substrates, panel-level processing, beam shaping, multi-beam systems, and design-process reliability co-optimization. Application drivers include 2.5D/3D packaging, MEMS, RF modules, optoelectronics, co-packaged optics, AI chips, and 6G/terahertz systems.

## Figures and Tables

**Table 1:** Classification and process characteristics of representative TGV methods [14,15,16,17,18,19].

Category	Method	Processing Principle	Main Features	Typical Applications
Non-laser TGV methods	Sandblasting erosion	Glass is removed by abrasive particle impact	Low cost; suitable for batch processing	Low-cost glass via fabrication; preliminary via forming
	Mechanical drilling	Glass is removed by microdrilling tools	Mature process; simple equipment; direct hole forming	Thick glass substrates; low-density via structures
	Photosensitive glass method	UV exposure and heat treatment enable selective etching of modified regions	Good patternability; suitable for fine microstructures	MEMS glass substrates; microfluidic devices
	Electrochemical discharge machining	Local discharge and electrochemical reaction remove glass material	Suitable for insulating brittle materials; flexible hole machining	Micro-holes in glass; special-shaped via fabrication
	Deep reactive ion etching	Plasma etching removes glass through anisotropic dry etching	Good dimensional control; vertical sidewalls possible	High-precision MEMS structures; glass microvias
	Wet etching	Chemical solution dissolves glass through mask-defined regions	Low cost; batch processing; simple operation	Glass microchannels; shallow via structures
Laser-based TGV methods	CO_2_ laser ablation	Thermal laser energy melts and evaporates glass	High efficiency; suitable for thick glass processing	TGV drilling; glass interposer fabrication
	Femtosecond/picosecond laser ablation	Ultrashort pulses induce nonlinear absorption and precise material removal	High precision; small heat-affected zone; flexible patterning	High-density TGVs; precision glass micromachining
	Laser-induced deep etching	Laser modification followed by selective wet etching forms deep vias	High aspect ratio; good sidewall quality; suitable for wafer-scale processing	Glass interposers; wafer-level packaging
	Femtosecond laser-assisted etching	Femtosecond laser modification enhances selective chemical etching	High precision; suitable for complex 3D glass structures	Microfluidic chips; 3D glass microstructures
	QCW laser drilling	Quasi-continuous-wave laser removes glass mainly by thermal drilling	High processing speed; suitable for mass production	High-throughput TGV fabrication; advanced packaging

**Table 2:** Comparison of key performance indicators for non-laser TGV methods.

Processing Method	Hole Quality (Diameter/Aspect Ratio/Roughness)	Hole Profile Deviation/Taper	Metallization Quality	Key Thermomechanical Reliability Issues	Ref.
Sandblasting erosion	>200 μm, <1, Ra 0.32–0.75 μm	Severe taper, poor sidewall verticality	Shadowing effect causes seed layer discontinuity; high risk of filling voids	Surface microcracks serve as crack initiation sources for thermal shock fracture; delamination under thermal cycling	[11,21,22,23,24]
Mechanical drilling	>100 μm, ≤10 (ultrasonic), Ra 0.01–2 μm(RUM)	Entrance chipping, exit spalling	Rough sidewall affects seed layer uniformity	Chipping and subsurface damage serve as crack initiation sites for crack propagation	[25,26,27,28]
Photosensitive glass method	30–200 μm, 7:1–35:1, Ra < 1 μm	Hourglass shape, ~2.5° sidewall taper; 10–20 μm over-etch at openings	Smooth sidewall facilitates continuous seed layer deposition	CTE mismatch requires attention; high-temperature crystallization may introduce warpage	[29,30,31,32,33,34]
ECDM	200–580 μm, <5, —	HAZ 48–87 μm, overcut 73–160 μm	Resistance 256 mΩ (<±8%), dense filling	HAZ microcracks risk propagation under thermal cycling	[37,41,42,43,44]
DRIE	40–80 μm, >10, Ra ~ 4 nm	Bottom angle ≈ 88°, excellent verticality	Smooth surface serves as ideal metallization substrate	Minimal microcracks, intrinsically good reliability; limited by cost and throughput	[45,46]
Wet etching	Minimum diameter 25.68 μm, 0.3:1–8:1	Isotropic etching causes undercutting, sidewall tilt	Inclined sidewalls facilitate the sputter deposition of metal layers	High-temperature heat treatment prone to deformation; via position offset impairs signal integrity; mask microcracks may induce etching defects	[50,51,52]

**Table 3:** Comparison of key performance indicators for laser-based TGV methods.

Processing Method	Hole Quality (Diameter/Aspect Ratio/Roughness)	Hole Profile Deviation/Taper	Metallization Quality	Key Thermomechanical Reliability Issues	Ref.
CO_2_ laser ablation	<100 μm, —, —	Tapered profile, recast layer on sidewalls	Taper and rough sidewalls hinder continuous seed layer coverage	Residual thermal stress evolution during long-term thermal cycling requires attention	[56,57,58,59]
Femtosecond/picosecond laser ablation	<50 μm, >8:1, —	HAZ < 5 μm, nearly no recast layer	Low thermal damage benefits subsequent metallization	Residual stress may drive crack initiation or interface delamination during thermal cycling	[60,61,62,63]
LIDE	10–50 μm, up to 200:1, lower roughness than single process	Taper angle < 5°, sidewall verticality near 90°	Low roughness and vertical sidewalls provide ideal substrate for metallization	Processing below damage threshold; no microcracks; intrinsically excellent reliability	[66,67,68,69]
FLAE	Up to <60 μm, up to 200:1, —	Vertical sidewalls, controllable via nanograting mechanism	Vertical sidewalls and low roughness facilitate seed layer coverage and void-free filling	No thermal damage; no microcracks; excellent thermal cycling reliability	[75,76]
QCW laser drilling	10–50 μm, >10, —	Nearly taper-free; single-hole formation in 10–20 μs	High-aspect-ratio vias impose high requirements on seed layer continuity and filling capability	Residual stress from extremely short thermal shock requires further evaluation	[77]

**Table 4:** Comparison of different glass materials for TGV fabrication.

Glass Material	Main Advantages	Main Limitations	Suitable TGV Methods
Borosilicate glass	Good processability; widely used	Thermal stress and cracks need control	Laser drilling, LIDE, wet etching-assisted methods [75]
Fused silica	Low dielectric loss; high thermal stability	Difficult to machine; low etching rate	Fs/ps laser, FLAE, laser-assisted etching [43]
D263 glass	Thin substrate; good surface quality	Handling and stress control are difficult	LIDE, laser drilling, wet processing [57]
Photosensitive glass	Selective etching; smooth via profile	Material limitation; complex process	Exposure, heat treatment, wet etching [31]

**Table 5:** Quantitative comparison of key performance indicators for representative TGV fabrication methods.

Method	Via Diameter/AR	Sidewall Quality	Throughput	Process Cost	Scalability/Adoption Evidence	Ref.
Sandblasting	>100 μm; AR < 2	Rough; severe taper; chipping	High; batch wafer-level process	Low–medium	Coarse vias only; fine-pitch limited	[11,21,22,23,24,30]
Mechanical drilling/μRUM	≥100–300 μm; AR: 3–3.5	Ra0.01–2 μm; chipping/spalling possible; taper can be <0.1° after optimization	Low–medium; serial drilling	Low–medium	Suitable for large vias; tool wear limits mass production	[25,26,27,28]
ECDM	~200–580 μm; AR < 5	HAZ 48–87 μm; overcut 73–160 μm;	Medium; multi-tip array	Low	Cu-filled TGVs demonstrated; limited fine-pitch	[37,42,43,44]
DRIE/ICP-RIE	40–80 μm; AR > 10	Ra ~4 nm; near vertical; base angle ~88°	Low; ~0.6 μm/min	High	MEMS scale demonstrated; limited throughput	[45,46]
Wet etching	Tens to 100+ μm; AR low–medium	Smooth; isotropic undercut; angle ~45–50°	Batch but slow	Low–medium	Widely used for MEMS; poor high-AR capability	[51,52]
CO_2_ laser drilling	<100–200 μm; AR ~ 5	HAZ/recast possible; crack risk	High; ~0.25 s/hole	Medium	Mature; crack control required	[56,57,58,59]
Fs/ps laser ablation	<50–100 μm; AR ~ 8	Low HAZ; taper < 5° possible;	Medium; serial	High	Precision microvias; limited throughput	[60,61,62,63]
LIDE/SLE	~10–60 μm; high-AR feasible	No microcracks; near vertical; etch-dependent	Fast modification + etching	Medium	8-inch wafer demonstrated; 110 GHz, 128 Gbaud	[66,67,68,69,75,76,81,105]
QCW laser drilling	~10–20 μm; AR ~5–10	Smooth, straight; minimal splash	Very high; 10–20 μs/via	Medium–high	Thin-glass high throughput; reliability data limited	[77]

Note: AR = aspect ratio; HAZ = heat-affected zone; SLE = selective laser etching; LIDE = laser-induced deep etching; QCW = quasi-continuous wave; N.R. = not reported. μRUM = micro-rotary ultrasonic machining. Quantitative values are summarized from the cited literature. Process cost and scalability are evaluated qualitatively based on equipment complexity, throughput potential, and demonstrated industrial adoption.

## Data Availability

Data is available upon request.
